# Surface Integrity and Friction Performance of Brass H62 Textured by One-Dimensional Ultrasonic Vibration-Assisted Turning

**DOI:** 10.3390/mi12111398

**Published:** 2021-11-14

**Authors:** Xianfu Liu, Jianhua Zhang, Li Li

**Affiliations:** 1 School of Mechanical Engineering, Shandong University of Technology, Zibo 255000, China; sdutlili@163.com; 2 Key Laboratory of High Efficiency and Clean Mechanical Manufacture, Ministry of Education of China, School of Mechanical Engineering, Shandong University, Jinan 250061, China; jhzhang@sdu.edu.cn

**Keywords:** ultrasonic vibration-assisted turning, micro-textured surface, dimple profile, subsurface microstructure, friction performance

## Abstract

The processing method, one-dimensional ultrasonic vibration-assisted turning (1D UVAT), is a potential and efficient way for fabricating a micro-textured surface. This paper aims at exploring the surface integrity and friction performance of brass H62 textured by the 1D UVAT. Four micro-textured surfaces with a specific distribution, size, and shape of dimples were fabricated by optimizing processing parameters, and the corresponding surface topography, subsurface microstructure, and surface roughness were observed and analyzed. A series of friction tests were carried out under oil-lubricating conditions to research the friction performance of micro-textured surfaces. The results show that the reason for the deviation between theoretical and experimental values of dimple depth was further revealed by observing the corresponding subsurface microstructure. The surface roughness of the micro-textured surfaces prepared is related to the number of micro-dimples per unit area and dimple size, which is greater than the surface generated by conventional turning. Compared with the polished surface and micro-grooved surface, the micro-textured surfaces have better friction performance with a lower frictional coefficient (COF) and wear degree. For the micro-textured surface fabricated by 1D UVAT, the number of micro-dimples per unit area has a great effect on the friction performance, and choosing a larger number is more conducive to improving the friction performance under the oil-lubricating condition. Consequently, this study proves that the proposed 1D UVAT can be a feasible candidate for preparing a micro-textured surface with better tribological property.

## 1. Introduction

In recent years, due to the potential of micro-textures such as dimples, grooves, and convex areas to improve surface performance, the corresponding research and applications of micro-textured surfaces have gained more and more attention in the engineered fields [1,2,3,4]. For example, the specific micro-textures on the surface of tribo-pairs can greatly enhance the friction characteristics and load-carrying capacity under certain conditions, which is also beneficial to reduce the energy losses [5,6,7,8]. The fabricated micro-textures on the surface in the disk start–stop area can effectively reduce the adhesion and friction between the disc and head, which can further prolong the service life of the magnetic disc [9,10,11,12]. The special micro-textures on the tool surface can reduce the contact length between the tool and chip, store the lubricant, and improve the wear resistance of the cutting tool [13,14,15,16]. The micro-textured surface designed according to the biological surface of nature can realize self-cleaning, drag reduction, and other functions [17,18,19,20,21].

Many typical processing technologies have been applied to the preparation of micro-textured surfaces, such as laser surface texturing (LST) [6,7,22], chemical etching [23], micro-forming [24], electrical discharge texturing (EDT) [25], abrasive-jet machining (AJM) [26], LIGA [27], and elliptical vibration texturing [28,29]. Each existing method of surface texturing has its own processing characteristic and application range of materials. Among which, the LST is a more widely used method in the application field of micro-textured surface preparation, which has a fast laser texturing speed and relatively accurate control ability in the dimension and shape of micro-textures. However, for the LST, the “ablation” damage of the fabricated micro-textures can be inevitably produced due to the rapid melt and solidification process. For most of the current surface texturing technologies, it is still difficult for them to meet the requirement of the large-scale industrial production due to the complex processing mechanism, the relatively low processing efficiency, the expensive equipment, or other reasons. Therefore, it is still essential to develop relatively more simple, efficient, and cost-effective methods to meet the requirement of mass production [30].

When ultrasonic vibration is applied to the depth direction of turning, the special characteristic of cutting trace during the vibration cutting process can make the surface texturing possible, and the corresponding method is 1D UVAT. From the previous studies [31,32,33], it can be confirmed that 1D UVAT is a simple, feasible, and efficient method for fabricating continuous micro-dimples with specific distribution, size, and shape on the cylindrical and end surfaces. The previous studies mainly focused on the texturing process and mechanism of 1D UVAT through theoretical analysis, simulation prediction, and experimental verification. Except for the surface topography, the paper will more deeply carry out the research on the surface integrity and friction performance of brass H62 textured by the 1D UVAT, which can further reveal the texturing process and mechanism more comprehensively. The paper will provide the actual validation for the reason of deviation between theoretical and experimental values of dimple depth through the study of subsurface microstructures. Four micro-textured surfaces were firstly fabricated by optimizing the processing parameters. Then, the corresponding surface topography, microstructure, and roughness were analyzed. For the friction performance, a series of friction tests under oil lubricating condition were conducted on the brass surfaces. Through comparing and analyzing the COF and worn morphology of different surfaces including the polished surface, the micro-grooved surface (fabricated by conventional turning), and the four micro-textured surfaces (fabricated by 1D UVAT), the influences of micro-dimples on the surface friction performance were finally analyzed.

## 2. Sample Preparation

**Figure 1:**
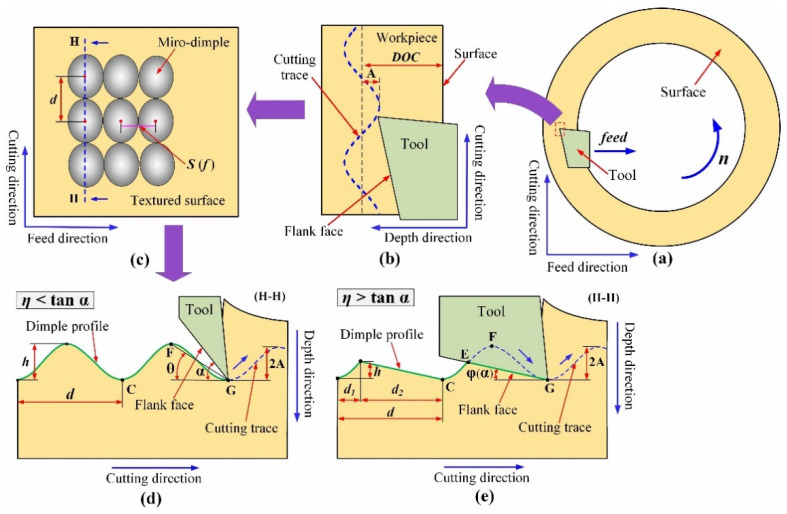
Illustration of the surface texturing process by 1D UVAT: (**a**) end face; (**b**) cutting process; (**c**) micro-textured surface; (**d**) dimple profile fabricated under *η* < tan*α*; (**e**) dimple profile fabricated under *η* > tan*α*.

The illustration of the surface texturing process by 1D UVAT can be seen in Figure 1. Considering that the workpiece used in the paper is ring-shaped, the schematic workpiece surface (end face) in Figure 1a is also in the shape of a ring. During the surface texturing process, the workpiece rotates with the spindle of turning, and the cutting tool not only moves along the feed direction but also makes ultrasonic vibration in the depth direction. The resulting cutting trace can be seen in Figure 1b. The depth of cut (*DOC*) chosen for the surface texturing process is larger than the vibration amplitude (*A*), and the tool can continuously cut the surface to fabricate the continuous micro-dimples in the cutting direction. The illustration of the distribution and geometry of micro-dimples on the surface is shown in Figure 1c. According to the previous study [33], the distance between two adjacent dimples in the cutting direction (*d*), the distance between two adjacent micro-dimples in the feed direction (*S*), and the dimple depth (*h*) can be controllably changed through choosing different processing parameters. Among them, *d* and *S* determine the number of micro-dimples per unit area. The distance between two adjacent dimples in the cutting direction can be calculated by the following:

(1) $$d=\frac{\pi Rn}{30f_{us}}$$

where *f_us_* is the vibration frequency, *R* represents the radius corresponding to the point on the end face, and *n* is the spindle speed. The distance between two adjacent micro-dimples in the feed direction is equal to the feed rate, which can be shown by the following:

(2) S=f

where *f* represents the feed rate. When ultrasonic vibration parameters are kept constant, the values of *d* and *S* are proportional to the spindle speed and feed rate, respectively.

As for the dimple depth and shape, the intersection state between the cutting trace and the flank face of the tool plays a key role. The corresponding detailed description can be seen in Figure 1d,e. Then, the angle between CG and FG is defined as *θ*, and the tangent of θ is defined as *η*. For the end face, when the tool is at the same point, choosing a different clearance angle (*α*) can make the intersection state change, resulting in the change of dimple depth and profile. When *η* is less than tan*α*, that is *η* < tan*α*, the flank face has no intersection with the cutting trace, and the corresponding dimple depth is theoretically equal to 2*A*, which can be formulated by the following:

(3) h=2A.

When *η* is greater than tan*α*, that is, *η* > tan*α*, the flank face intersects with the cutting trace at the point E. The angle between CG and EG is defined as *φ*, which is equal to *α*. Under this intersection state, the corresponding dimple depth and profile also change, and the *d* contains two distances *d*_1_ and *d*_2_ of different lengths. The dimple depth can be theoretically calculated by the following:

(4) $$h=\frac{\pi Rn}{30}(\frac{1}{f_{us}}-t_{1})\tan\varphi$$

where *t*_1_ represents the time taken for the tool from point C to point E. More corresponding detailed theoretical analysis and calculation processes can be found in the previous study [33]. Based on the above theoretical analysis, four micro-textured surfaces with different *d*, *S*, *h,* and shapes were prepared in the paper.

Brass is a metal composed by the main elements of copper and zinc, which is also known as the copper zinc alloy. As a result of its good processing performance, mechanical property, corrosion resistance, and high thermal conductivity, brass has become one of the most widely used non-ferrous metal materials, which can be applied for the preparation of mechanical or stamping parts. Therefore, the brass H62 is selected as the workpiece material for further research on the surface integrity and friction performance of micro-textured surfaces fabricated by 1D UVAT.

**Figure 2:**
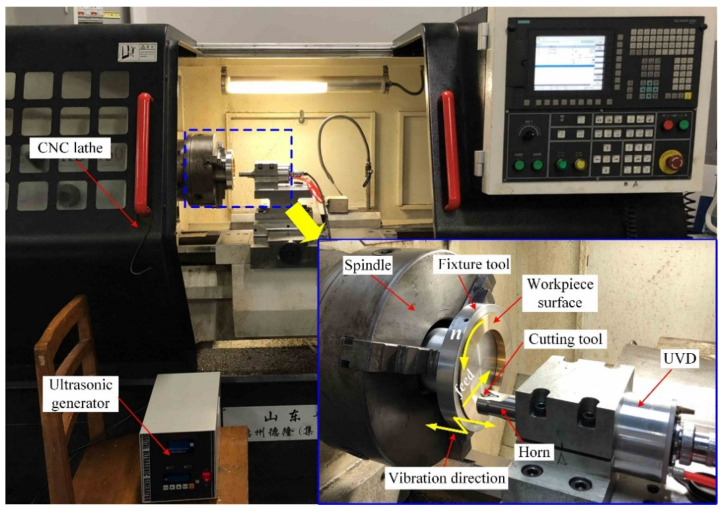
Experimental setup for generating the micro-textured surface of a brass H62 workpiece.

The experimental setup for generating the micro-textured surface of the brass H62 workpiece can be seen in Figure 2. The ultrasonic vibration device (UVD) is mounted on the sliding platform of CNC lathe (CKD6150H, Precion, China) through a base. An ultrasonic generator provides the power for the UVD. The ultrasonic amplitude and frequency of the UVD are respectively 3.9 μm and 19,670 Hz, which were measured by a laser Doppler vibrometer (OFV5055000, Polytec, Germany). A PCD (Polycrystalline Diamond) tool is fixed on the front of the horn through a screw, and it can make an ultrasonic vibration in the depth direction when the UVD works. For facilitating the preparation of subsequent test blocks, the shape and size of the brass workpiece are different from that of the workpiece processed in the previous paper [33]. The brass workpiece is ring-shaped, of which the outer diameter is 128 mm, the inner diameter is 92 mm, and the thickness is 12 mm. The ring-shaped workpiece is fixed in a specially designed fixture tool through three screws. The fixture tool is installed on the spindle through a chuck, which can drive the workpiece to rotate with the spindle. For the UVD, the ultrasonic amplitude and frequency are kept constant during the sample preparation. For the tool, the clearance angles are 20° and 7°, and the nose radii are 200 μm and 100 μm. As for the cutting parameters, the depth of cut is 80 μm, the spindle speed is 300 r/min and 600 r/min, and the feed rate is 110 μm/rev and 80 μm/rev.

Referring to previous research results, the optimized combination of processing parameters (from entry #1 to entry #4) used for fabricating the specific four micro-textured surfaces are summarized in Table 1. For the convenience of the subsequent analysis and description, the micro-textured surfaces corresponding the processing parameters of entries #1, #2, #3, and #4 are respectively defined as surface #1, surface #2, surface #3, and surface #4 in turn. As an experimental comparison, two other surfaces were also prepared. One is a surface fabricated by the turning on the same experimental platform without ultrasonic vibration, and the corresponding processing parameters were listed in entry #5 of Table 1. Except for the vibration parameters, the other processing parameters are the same as entry #1. The surface is defined as surface #5. The other surface is a polished surface, which is defined as surface #6.

**Figure 3:**
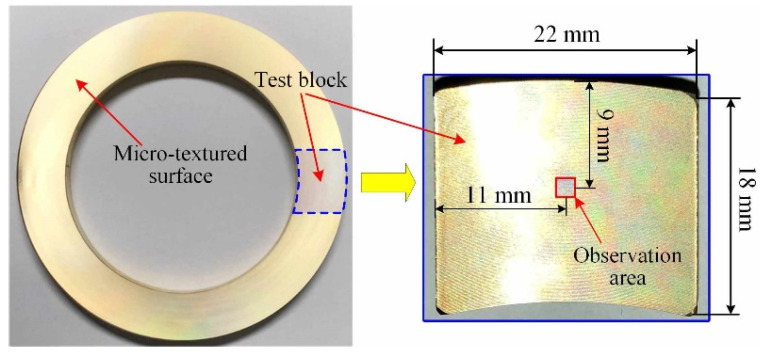
Preparation of the test block.

After finishing the surface texturing, a test block on which it is easy to carry out the following surface observation and friction test needs to be cut from the workpiece, as shown in Figure 3. The length, width, and height of the test block are 22 mm, 18 mm, and 12 mm, respectively. For the end face, the size and shape of micro-dimples on different positions in the feed direction can change [33]. In order to avoid the influence of the end face position on micro-dimples, the observation area of all surfaces is set in the middle of the test block surface (the red box area in Figure 3) and remains unchanged.

## 3. Surface Topography

**Figure 4:**
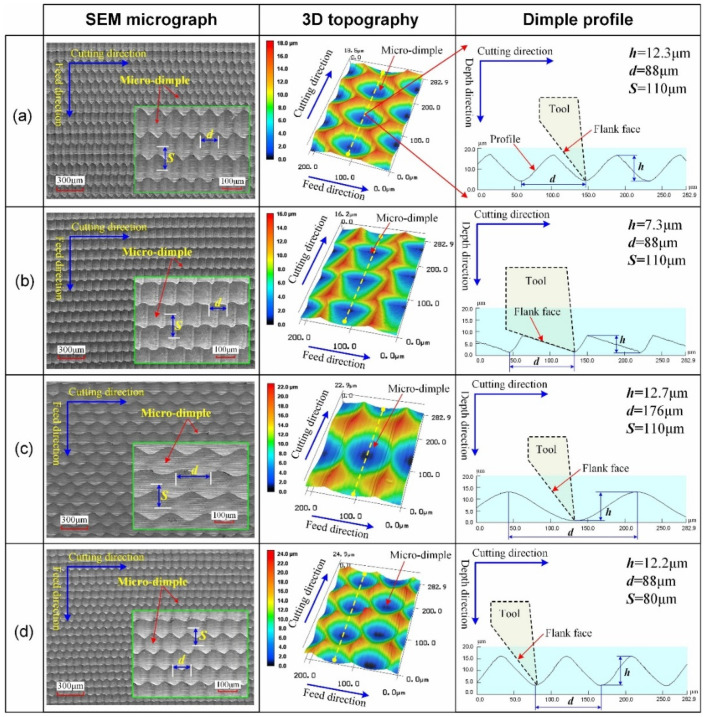
Surface topography of the four micro-textured surfaces fabricated by 1D UVAT: (**a**) surface #1; (**b**) surface #2; (**c**) surface #3; (**d**) surface #4.

In order to analyze the surface topography, a scanning electron microscope (SUPRA™ 55, Zeiss, Germany) was used to take micrographs, and a laser microscope (VK-X200, Keyence, Japan) was used to observe the 3D topography and dimple profile. The detailed SEM micrograph, 3D topography, and dimple profile of the four micro-textured surfaces are shown in Figure 4. It can be found that all the micro-textured surfaces were covered with continuous dimples, and then, they can also be called the micro-dimpled surface. Surfaces #1, #3, and #4 were processed under the condition of *η* < tan*α*, and surface #2 was processed under the condition of *η* > tan*α*. The dimple shape of surfaces #1, #3, and #4 is oval-like, and the corresponding dimple profiles all present a sinusoidal curve. Different from the above three surfaces, the dimple shape of surface #2 is scale-like, and an angle *φ* exists in the dimple profile due to the existence of an intersection between the cutting trace and flank face. For surface #1, the values of *h*, *d*, and *S* are respectively 12.3 μm, 88 μm, and 110 μm. Surface #2 has a smaller dimple depth compared with surface #1, and the corresponding values of *h*, *d*, and *S* are respectively 7.3 μm, 88 μm, and 110 μm. For surface #3, it has a larger distance between two adjacent micro-dimples in the cutting direction compared with surface #1, and the corresponding values of *h*, *d*, and *S* are respectively 12.7 μm, 176 μm, and 110 μm. For surface #4, it has a smaller distance between two adjacent micro-dimples in the feed direction compared with the surface #1, and the corresponding values of *h*, *d,* and *S* are respectively 12.2 μm, 88 μm, and 80 μm. As for the number of micro-dimples per unit area, the number for surface #3 is the smallest, the number for surface #4 is the largest, and the number for surface #1 is equal to that of surface #2. The corresponding main parameters of micro-dimples on the surfaces are summarized in Table 2.

**Figure 5:**
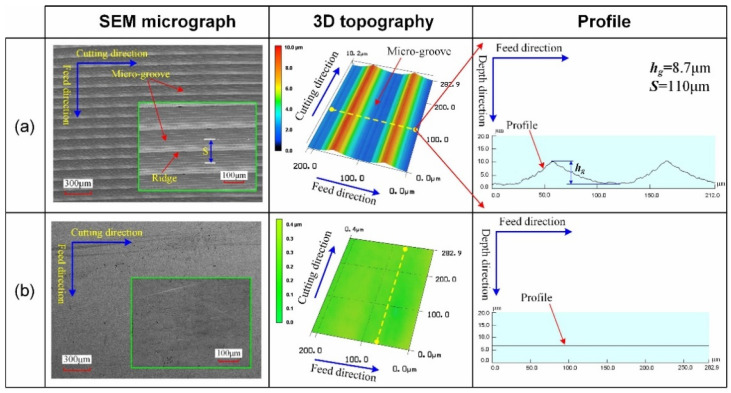
Surface topography of micro-grooved surface and polished surface: (**a**) surface #5; (**b**) surface #6.

The detailed SEM micrograph, 3D topography, and profile of surfaces #5 and #6 can be seen in Figure 5. For surface #5, it is found that the micro-grooves are evenly distributed on the surface. Therefore, surface #5 is also called a micro-grooved surface. The distance between two adjacent micro-grooves in the feed direction is 110 μm, and the depth of the micro-groove (*h_g_*) is measured to be 8.7 μm.

## 4. Subsurface Microstructure

**Figure 6:**
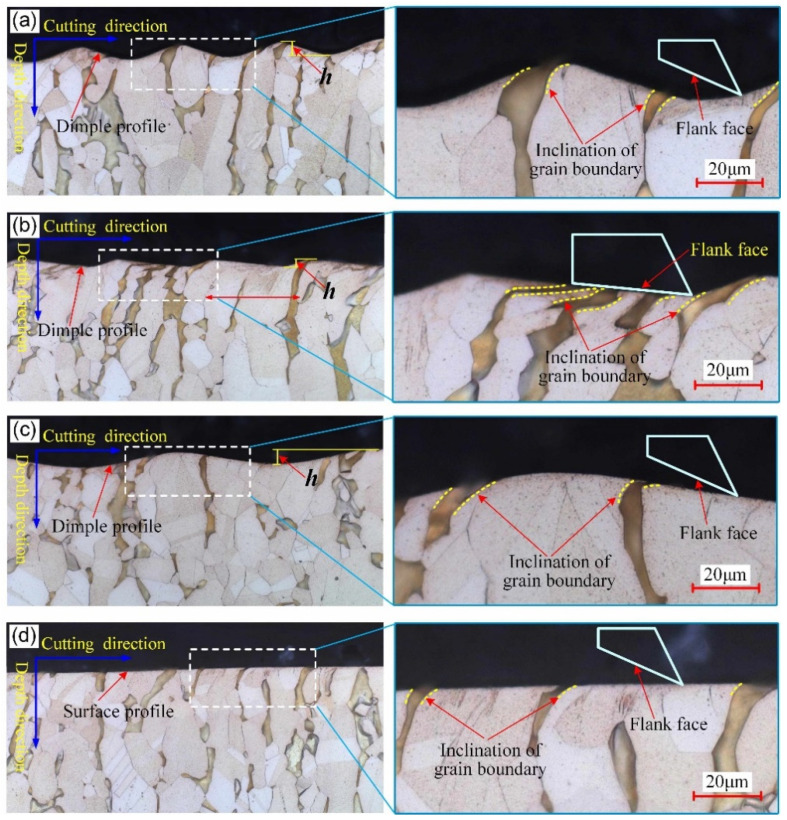
Subsurface microstructure of different surfaces: (**a**) surface #1; (**b**) surface #2; (**c**) surface #3; (**d**) surface #5.

In order to observe subsurface microstructure, the middle position of the test block surface was first cut along the cutting direction to prepare the inlaid specimen. The cross-section of the inlaid specimen was polished by an automatic vibration polishing machine, and then, the polished section was corroded by ferric chloride hydrochloric acid alcohol solution. A metallurgical microscope (Axio Oberver A1m, Zeiss, Germany) was used to observe the subsurface microstructure, and the corresponding observation results of surfaces #1, #2, #3, and #5 can be seen in Figure 6. Since the dimple profile of surface #1 is basically the same as that of surface #4 and the corresponding cutting process in the cutting direction is consistent, the subsurface microstructure of surface #4 was no longer observed.

From Figure 6, it can be seen that the inclination degree and depth of the grain boundary of micro-textured surfaces #1, #2, and #3 are all greater than those of micro-grooved surface #5. The corresponding inclination direction is all along the cutting direction. For surfaces #1 and #3, the dimple profile corresponding to the subsurface presents a sinusoidal curve, which is same as that of the dimple profile derived from the observed 3D topography. Under the intersection state of *η* < tan*α*, the instantaneous extrusion of the surface material by the cutting edge and the shear stress due to friction between the tool and workpiece cause the plastic deformation and shear slip of the subsurface metal grains, resulting in the occurrence of elongation of grain and inclination of the grain boundary during the vibration cutting process, as shown in Figure 6a,c. It confirms that the elastoplastic deformation of the material is real in this surface texturing. This contributes to revealing the phenomenon that the actual dimple depth is larger than the theoretical predicted value of 7.8 μm. When the intersection state is *η* > tan*α*, the corresponding subsurface microstructure of surface #2 can be seen in Figure 6b. The dimple profile corresponding to the subsurface is also same with that derived from the observed 3D topography. In particular, both the elongation of grains and inclination of grain boundaries are the most serious compared with those of surfaces #1 and #3. It is further proved that when the flank face intersects with the cutting trace, the instantaneous extrusion from the flank face is greatly enhanced, leading to the enhancement of plastic deformation of the subsurface. This also contributes to revealing the phenomenon that the actual dimple depth is larger than the theoretical predicted value of 6.7 μm.

From the above analysis, it is further confirmed that when the ultrasonic vibration is applied to the depth direction of turning, the elastoplastic deformation of the subsurface becomes larger, and the intersection state between the flank face and cutting trace has an important effect on the deformation degree.

## 5. Surface Roughness

**Figure 7:**
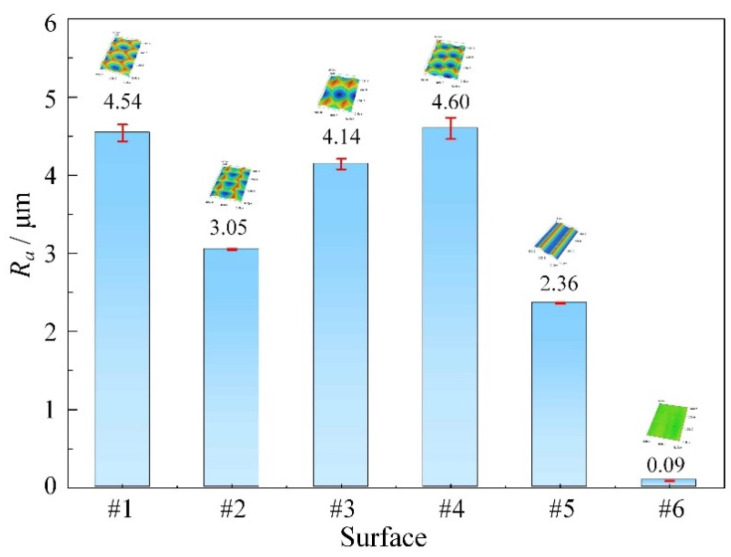
Surface roughness of different surfaces.

The laser microscope (VK-X200, Keyence, Japan) was used to measure the surface roughness (*R_a_*) for surfaces #1–#6. The corresponding scanning area is the same with the observation area, as shown in Figure 3. Three points were measured on every surface, and the average value was taken as the value of the surface roughness. The measured results of the surface roughness are shown in Figure 7. It can be found that the roughness of the micro-textured surfaces fabricated by 1D UVAT is greater than that of the micro-grooved surface processed by the conventional turning. The roughness from surface #1 to surface #4 is 4.54 μm, 3.05 μm, 4.14 μm, and 4.60 μm, respectively. Among the four micro-textured surfaces, surface #2 with a minimum dimple depth fabricated under the intersection state of *η* > tan*α* shows the lowest roughness. For surfaces #1, #3, and #4 fabricated under the intersection state of *η* < tan*α*, the differences in the number of micro-dimples per unit area and dimple size also lead to the change of the corresponding surface roughness.

## 6. Friction Performance

### 6.1. Test Procedure

**Figure 8:**
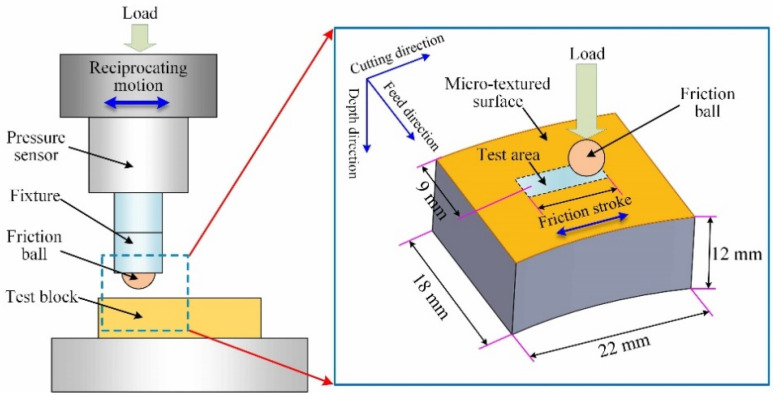
Principle diagram of the test mode of ball-on-disk.

A universal micro-tribotester (UMT-2, CETR, Bruker, Billerica, MA, USA) was used for the friction tests, which can collect in real time and output the change of COF during the test processing. The test mode of ball-on-disk was adopted, and the corresponding principle diagram can be found in Figure 8. The test block is fixed on the platform and the micro-tribotester drives the friction ball to carry out linear reciprocating movement on the sample surface under a certain load and speed. The test area is located in the middle of the sample surface. The load, speed, stroke, and time of the test can be set by the software.

**Figure 9:**
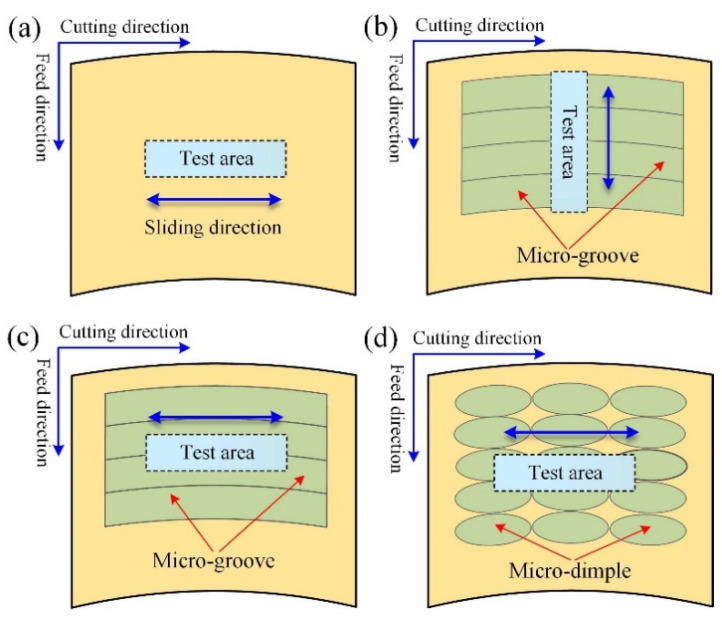
Test scheme for different surfaces: (**a**) surface #6; (**b**) surface #5; (**c**) surface #5; (**d**) surface #1.

Figure 9 shows the test scheme for different surfaces. For the micro-dimpled surface, since the micro-dimples are evenly distributed on the surface, only one test direction is selected. For the polished surface and micro-dimpled surface, the only the ball slides in the cutting direction, as shown in Figure 9a,d. For the micro-grooved surface, in order to analyze the friction performance more comprehensively, two obvious different test paths were selected. One test path is along the cutting direction, that is, the test direction is basically parallel to the micro-groove, as shown in Figure 9c. The other test path is along the feed direction, that is, the test direction is perpendicular to the micro-groove, as shown in Figure 9b. During the test processing, the stroke, time, speed, and load is 8 mm, 30 min, 6 mm/s, and 12 N, respectively. Each group under the same conditions is tested once. The tests were carried out under the condition of oil lubrication and room temperature. The selected lubricant was high-temperature lubricating oil, which can be used from the minimum temperature −30 °C to the maximum temperature +300 °C. Before the test, adequate lubricating oil was evenly dropped on the test area through a dropper, so that the tribo-contact surface can be immersed in oil during the test processing. A friction ball made of 440C stainless steel with a Rockwell hardness of 58HRC was used in this test. The diameter of ball is 9.525 mm. The ball surface is polished, and the surface roughness is about 0.02 μm. Before each test, it is necessary to replace the friction ball and install a new one. In addition, the test block should be cleaned by alcohol solution in an ultrasonic cleaning machine to remove surface impurities. After the test, the sample surface should be cleaned successively in acetone and alcohol solution using the ultrasonic cleaning machine, and then, the surface observation can be carried out.

### 6.2. Results and Discussion

**Figure 10:**
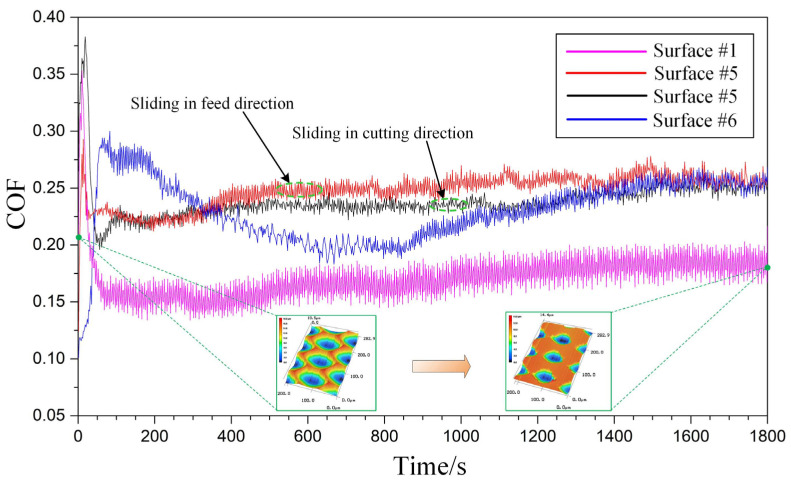
Variation curves of COF corresponding to different surfaces.

For surfaces #1, #5, and #6, the corresponding variation curves of COF within 30 min can be seen in Figure 10. The pink curve represents the COF of micro-dimpled surface #1, the red curve represents the COF of micro-grooved surface #5 sliding in the feed direction, the black curve represents the COF of micro-grooved surface #5 sliding in the cutting direction, and the blue curve represents the COF of polished surface #6. It can be seen that the curves of COF for the four surfaces all go through a running-in stage and then enter a stable stage. For the COF of polished surface #6, it takes a relatively long time to enter the stable stage, which is different from that of the micro-grooved surfaces. When the test time goes on for about 25 min, the curves of COF for the polished surface and micro-grooved surface gradually tend to be consistent. Through measuring the COF in the stable stage, the average values of surface #1, surface #5 (sliding in the feed direction), surface #5 (sliding in the cutting direction), and surface #6 are 0.170, 0.254, 0.237, and 0.232, respectively. The COF of micro-dimpled surface #1 is obviously smaller than that of the micro-grooved surface and polished surface, which can be respectively reduced by 33.1%, 28.3%, and 26.7%. Compared with the micro-grooved surface, when ultrasonic vibration is applied to the depth direction of turning under the same processing parameters, the specific continuous micro-dimples fabricated on the surface can greatly improve the friction performance. As shown in Figure 10, the previous continuous micro-dimples in the cutting direction have changed into the discrete dimples after the friction test, and the flat part between the residual dimples corresponds to the worn surface.

**Figure 11:**
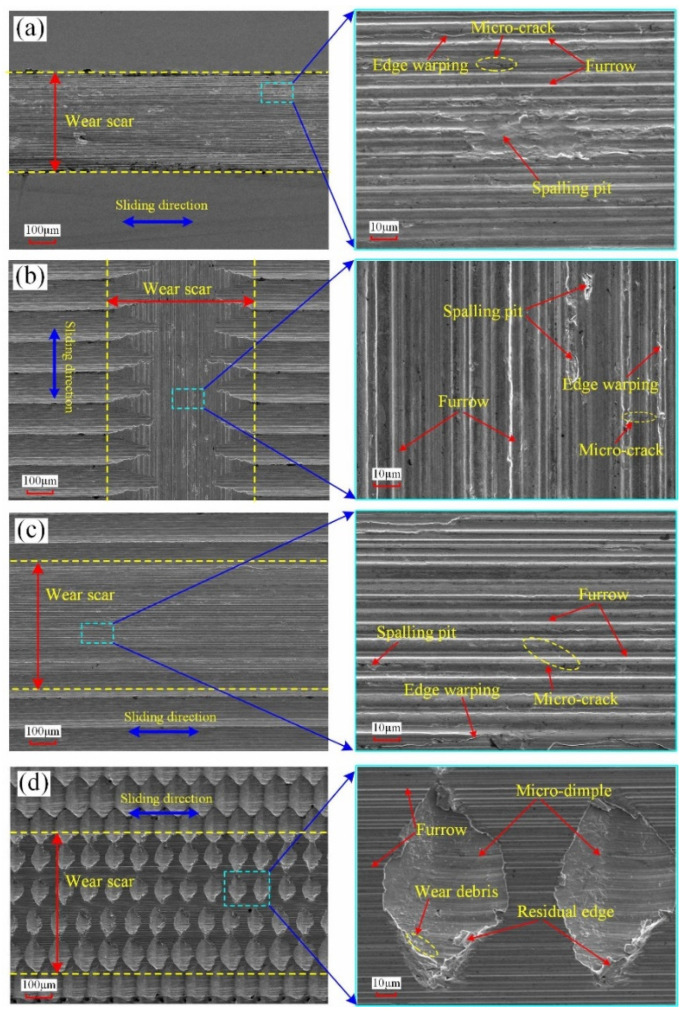
SEM micrographs of surface worn morphology: (**a**) surface #6; (**b**) surface #5 sliding in the feed direction; (**c**) surface #5 sliding in the cutting direction; (**d**) surface #1.

In order to further analyze the wear behaviors of the four surfaces during the sliding process, the corresponding SEM micrographs of surface worn morphology were observed, which can be seen in Figure 11. It can be found that the four surfaces all have a certain degree of wear. For the two micro-grooved surfaces sliding in different directions, the micro-grooves with a depth of 8.7 μm in the middle of the test area were totally smoothed out, and the relevant wear state is basically the same as that of the polished surface, as shown in Figure 11a–c. The furrow, spalling pit, micro-crack, and edge warping all exist on the worn area of surfaces #5 and #6. In addition, by observing the friction balls corresponding to the different worn surfaces, brass materials all adhere to the tribo-surface of the friction ball to varying degrees. It can be inferred that the adhesive wear, abrasive wear, fatigue wear, and plastic deformation probably exist in the sliding process. As can be seen from Figure 10, for polished surface #6, the COF increases first; then, it gradually decreases and then continues to gradually increase to the stable stage. In the initial friction contact process, the COF is small due to the existence of oxide film and boundary lubrication film on the polished surface. As a result of the contact of convex peaks, the oxide film is destroyed, and the COF increases rapidly. As the reciprocating sliding process progresses, the boundary lubrication film between the tribo-contact surface tends to be stable, and the COF tends to gradually decrease. However, as the reciprocating sliding process continues, there is a certain degree of adhesive wear between the tribo-contact surface, and the brass materials adhere to the surface of the friction ball, which leads to the increase in wear debris. Under the action of reciprocating contact stress, the wear debris has a greater ploughing effect on the surface, resulting in the furrows and edge warping. The boundary lubrication film is gradually destroyed to a certain extent. In addition, the worn area begins to appear micro-cracked, even the spalling pit, which leads to the increase in COF. Finally, the tribo-contact surface reaches the stable stage, which is the same as that of the micro-grooved surface. For surface #1, although a certain degree of surface wear also exists in the test area, there are still some residual micro-dimples with a regular size and uniform distribution on the surface, as shown in Figure 11d. The worn area of surface #1 mainly has a shallow furrow, and the spalling pit and micro-cracks have not been produced in the sliding process. Therefore, the wear degree of surface #1 is much smaller than that of the polished surface and micro-grooved surface. It shows that the continuous micro-dimples fabricated by 1D UVAT play a positive role in reducing the wear under the oil lubrication with low speed and light load. On the one hand, the evenly distributed micro-dimples have a good ability of oil storage in the reciprocating sliding process, which can continuously provide enough lubricating oil for the contact part of the tribo-surface to reduce the frictional resistance. Even if the supply of lubricating oil is not timely, the lubricating oil stored in the residual micro-dimples can make the tribo-surface be in a self-lubricating state within a certain time. On the other hand, as shown in Figure 11d, the wear debris produced in the reciprocating sliding process can be captured and contained by the micro-dimples, which is helpful to reduce the wear degree caused by abrasion. In addition, through observing Figure 11d, it is found that the part of the residual edge is depressed into the micro-dimples. Therefore, the micro-dimples can also store the residual edge to avoid the aggravation occurrence of plastic deformation and wear caused by the entrance of broken parts into the tribo-contact surface.

**Figure 12:**
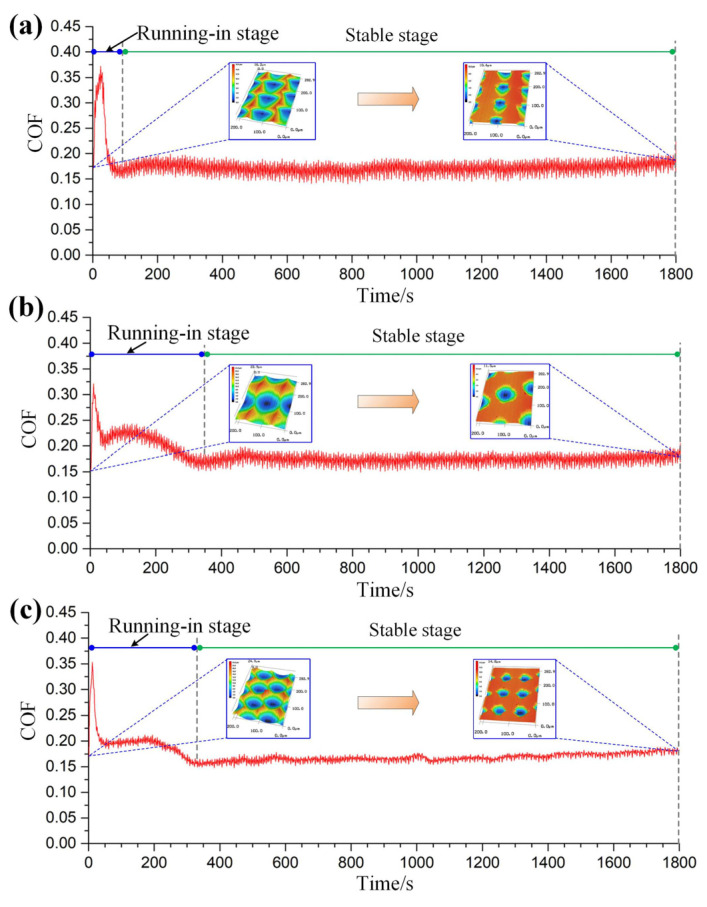
Variation curves of COF corresponding to the other three micro-textured surfaces: (**a**) surface #2; (**b**) surface #3; (**c**) surface #4.

**Figure 13:**
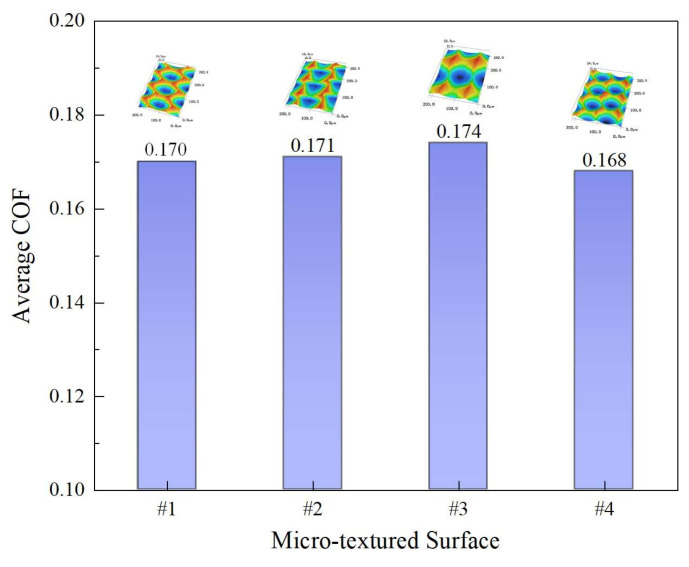
Average COF of the four micro-textured surfaces.

**Figure 14:**
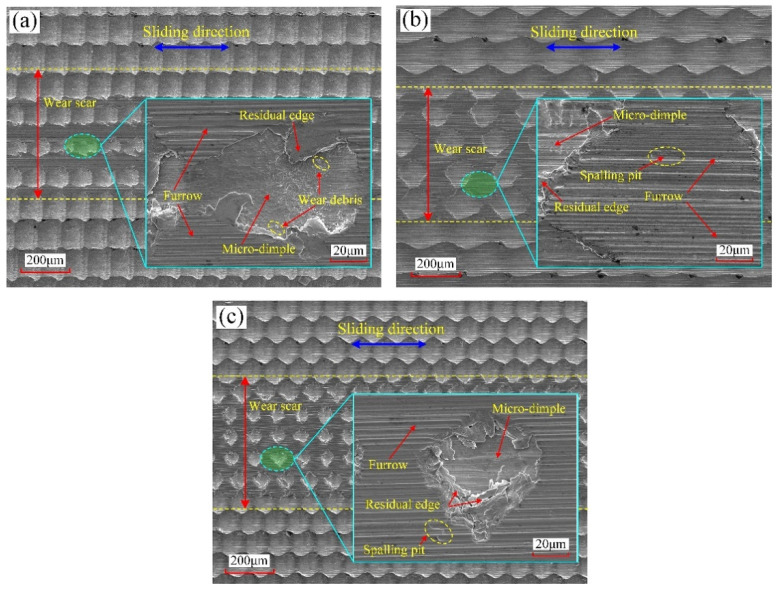
SEM micrographs of surface worn morphology: (**a**) surface #2; (**b**) surface #3; (**c**) surface #4.

Under the same test conditions, the COF curves and 3D topographies of the worn area corresponding to surfaces #2, #3, and #4 can be respectively seen in Figure 12. The three micro-dimpled surfaces all have undergone the running-in stage and then finally reached the stable stage during the sliding process. The residual micro-dimples are also evenly distributed on the worn surface. As shown in Figure 13, the average values of the COF in the stable stage corresponding to surfaces #2, #3, and #4 are 0.171, 0.174, and 0.168, respectively. Compared with surface #1, the average COF and the curve fluctuation degree of surface #2 have little change. As for the micro-dimples fabricated by 1D UVAT, the dimple shapes (scale-like and oval-like) and dimple depth have little effect on the friction performance under the current test conditions. In order to further observe the wear behaviors of the three surfaces during the sliding process, the corresponding SEM micrographs of surface worn morphology can be seen in Figure 14. For surface #2, the main form of surface wear is furrow. For the surfaces #3 and #4, except for the furrow, a spalling pit is also produced on the worn area. Among them, the furrow produced on surface #3 is more obvious. Due to the increase in distance between two adjacent dimples in the cutting direction, the number of micro-dimples involved in the storage of lubricating oil and debris is reduced, which leads to a certain enhancement in the degree of abrasive wear and plastic deformation. Consequently, the average COF of surface #3 in a stable stage becomes larger than that of surface #1. For surface #4, as the distance between two adjacent dimples in the feed direction decreases, the number of micro-dimples involved in the storage of lubricating oil and debris is increased. As a result, the friction performance of surface #4 has been improved, and the corresponding average value and fluctuation range of COF are also decreased accordingly. The above analysis of the friction performance mainly focuses on the effect of the surface micro-textures. Referring to the previous studies [34,35], the plastic strain and grain refinement produced in the subsurface can lead to the work-hardening, which is beneficial for the friction performance. By observing the subsurface microstructure in Figure 6, it can be inferred that the elastoplastic deformation and corresponding elongation of grains may also cause the surface hardening, which may be beneficial for the friction performance of the micro-textured surface. Therefore, in addition to the surface texturing effect, the potential effect of surface hardening on the friction performance should be considered in future research.

## 7. Conclusions

Based on the results and analysis in the present paper, the following conclusions can be drawn.

(1) By observing the inclination of grain boundary, it is confirmed that when the ultrasonic vibration is applied to the depth direction of turning, the elastoplastic deformation of the subsurface becomes larger, and the intersection state between the flank face and cutting trace has an important effect on the deformation degree. This provides a valid proof to further reveal the reason for the deviation between the theoretical and experimental values of dimple depth.
(2) Compared with the micro-grooved surface fabricated by the turning without ultrasonic vibration, the micro-textured surface generated by 1D UVAT has greater surface roughness. The surface roughness value is related to the number of micro-dimples per unit area and dimple size.
(3) Under the oil lubricating conditions of low speed and light load, the micro-dimpled surfaces fabricated by the 1D UVAT have better friction performance than the micro-grooved surface and the polished surface. Compared with the micro-grooved surface and polished surface, the average COF in the stable stage of the micro-dimpled surface can be reduced by up to 33.1%. The continuous micro-dimples mainly play the role of oil storage and debris containment in this linear reciprocating sliding process, which can provide enough lubricating oil for the tribo-surface and reduce the wear degree of the surface materials caused by abrasive. The number of micro-dimples per unit area has a great effect on the friction performance. Choosing the surface with a greater number of continuous micro-dimples per unit area is beneficial to improve the surface friction performance. As a consequence, this study proves that the proposed surface texturing method, 1D UVAT, can be a feasible candidate for the fabrication of micro-textured surfaces with better tribological property, which also lays a certain foundation for the further application of this surface texturing.

## Figures and Tables

**Table 1:** Processing parameters used for preparing surfaces.

#	Workpiece	Cutting Tool			Vibration Parameters		Cutting Parameters		
	Material	Material	Clearance Angle (°)	Nose Radius (μm)	Ultrasonic Frequency (Hz)	Vibration Amplitude (μm)	Depth of Cut (μm)	Spindle Speed (r/min)	Feed Rate (μm/rev)
1	H62	PCD	20	200	19,670	3.9	80	300	110
2	H62	PCD	7	200	19,670	3.9	80	300	110
3	H62	PCD	20	200	19,670	3.9	80	600	110
4	H62	PCD	20	100	19,670	3.9	80	300	80
5	H62	PCD	20	200	0	0	80	300	110

**Table 2:** The main parameters of micro-dimples on the surface.

Micro-Textured Surface	Parameters		
	*h*	*d*	*S*
#1	12.3 μm	88 μm	110 μm
#2	7.3 μm	88 μm	110 μm
#3	12.7 μm	176 μm	110 μm
#4	12.2 μm	88 μm	80 μm
